# Inhibition of TFF3 Enhances Sensitivity—and Overcomes Acquired Resistance—to Doxorubicin in Estrogen Receptor-Positive Mammary Carcinoma

**DOI:** 10.3390/cancers11101528

**Published:** 2019-10-10

**Authors:** Han Ming Poh, Yi Shiou Chiou, Qing Yun Chong, Ru-Mei Chen, Kanchugarakoppal S. Rangappa, Lan Ma, Tao Zhu, Alan Prem Kumar, Vijay Pandey, Basappa, Soo-Chin Lee, Peter E. Lobie

**Affiliations:** 1 Cancer Science Institute of Singapore, National University of Singapore, Singapore 117599, Singapore; han_ming89@hotmail.com (H.M.P.); qychong89@gmail.com (Q.Y.C.); chenrumei@u.nus.edu (R.-M.C.); csiapk@nus.edu.sg (A.P.K.); csilsc@nus.edu.sg (S.-C.L.); 2 Department of Pharmacology, Yong Loo Lin School of Medicine, National University of Singapore, Singapore 117600, Singapore; 3 Tsinghua-Berkeley Shenzhen Institute, Tsinghua University, Shenzhen 518055, Chinamalan@sz.tsinghua.edu.cn (L.M.); vijaypandey7@hotmail.com (V.P.); 4 Institution of Excellence, University of Mysore, Mysore 570005, India; rangappaks@yahoo.com; 5 Shenzhen Bay Laboratory, Shenzhen 518055, China; 6 Hefei National Laboratory for Physical Sciences at Microscale and School of Life Sciences, University of Science and Technology of China, Hefei 230027, China; zhut@ustc.edu.cn; 7 National University Cancer Institute, Singapore 119074, Singapore; 8 Cancer Program, Medical Science Cluster, Yong Loo Lin School of Medicine, National University of Singapore, Singapore 117597, Singapore; 9 Department of Studies in Organic Chemistry, University of Mysore, Mysore 570005, India; salundibasappa@gmail.com; 10 Department of Haematology-Oncology, National University Health System, Singapore 119228, Singapore

**Keywords:** mammary carcinoma, trefoil factor 3 (TFF3), doxorubicin, dose-dependent toxicity, acquired resistance, apoptosis, PI3K/AKT

## Abstract

Dose-dependent toxicity and acquired resistance are two major challenges limiting the efficacious treatment of mammary carcinoma (MC) with doxorubicin. Herein, we investigated the function of Trefoil Factor 3 (TFF3) in the sensitivity and acquired resistance of estrogen receptor positive (ER+) MC cells to doxorubicin. Doxorubicin treatment of ER+MC cells increased TFF3 expression. The depletion of TFF3 by siRNA or inhibition with a small molecule TFF3 inhibitor (AMPC) synergistically enhanced the efficacy of doxorubicin in ER+MC through the suppression of doxorubicin-induced AKT activation and enhancement of doxorubicin-induced apoptosis. Elevated expression of TFF3 and increased activation of AKT were also observed using a model of acquired doxorubicin resistance in ER+MC cells. AMPC partially re-sensitized the doxorubicin resistant cells to doxorubicin-induced apoptosis. Indeed, doxorubicin resistant ER + MC cells exhibited increased sensitivity to AMPC as a single agent compared to doxorubicin sensitive cells. In vivo, AMPC attenuated growth of doxorubicin sensitive ER+MC xenografts whereas it produced regression of xenografts generated by doxorubicin resistant ER+MC cells. Hence, TFF3 inhibition may improve the efficacy and reduce required doses of doxorubicin in ER+MC. Moreover, inhibition of TFF3 may also be an effective therapeutic strategy to eradicate doxorubicin resistant ER+MC.

## 1. Introduction

Despite the advances in MC treatment, the use of conventional chemotherapies, particularly those containing anthracyclines, remains important in the treatment of all MC subtypes [1] Indeed, neo-adjuvant chemotherapy remains the standard care for patients with locally advanced MC [2], where the use of an anthracycline containing regimen achieved a pathological complete response of 17.1% [3]. As an adjuvant therapy, the use of anthracycline containing regimens also significantly improved the 10-year recurrence free survival by 8% as well as produced a 5% reduction in MC mortality [4]. Doxorubicin is one of the most widely used anthracyclines for the treatment of MC [5]. However, the effective use of doxorubicin is limited by its dose-dependent cardiotoxicity and evidence of doxorubicin-related congestive heart failure have been reported in doxorubicin treated patients [6,7,8,9,10]. Furthermore, it is also limited by the acquired doxorubicin resistance that can be driven by various mechanisms including the upregulation of efflux transporters, enhanced DNA damage repair as well as the suppression of downstream apoptotic signaling [11]. Therefore, the development of potential combinatorial approaches to enhance the efficacy of doxorubicin without incurring further toxicity, and to overcome acquired doxorubicin resistance, is warranted. 

Trefoil factor 3 (TFF3), a member of the trefoil factor family, is a secreted protein expressed predominantly in the small and large intestine to promote cell migration and facilitate epithelial restitution upon gastrointestinal injury [12]. It contains a trefoil domain, and a cysteine residue (Cys-57) in the carboxy-terminal that facilitates its homodimerization essential for its anti-apoptotic and pro-proliferative functions [12,13,14,15]. Aberrant expression of TFF3 has been reported in various cancers including those of the breast [16,17,18], endometrium [19], prostate [20,21], cervical [22], liver [23], colon [24], gastric [25] and lung [26]. In recent years, a substantial amount of evidence that supports the diverse oncogenic roles of TFF3, including promotion of proliferation and survival [17,27], migration and invasion [16,28], and angiogenesis [29,30], have been reported. Clinically, TFF3 expression is associated with poor survival outcomes in MC patients [16]. In the context of drug sensitivity and resistance, lower serum TFF3 is correlated to a better chemotherapeutic response in gastric and colorectal cancer patients [31]. TFF3 has also been reported to mediate both tamoxifen [17] and trastuzumab resistance [32] in human mammary carcinoma, as well as doxorubicin resistance in hepatocellular carcinoma [23]. Consistently, an earlier study reported that the depletion of TFF3 sensitizes gastric cancer cells to doxorubicin-induced apoptosis [33]. Hence, pharmacological inhibition of TFF3 in cancer, either as a monotherapy or in combination with standard of care therapies, may be considered. 

TFF3 has been reported to bind LINGO2 [34], CXCR4/7 and other as yet unidentified receptors [35]. In addition, TFF3 indirectly activates other receptors including the HER family of tyrosine kinases and insulin-like growth factor receptor 1 (IGFR1) [32], triggering a multitude of downstream pathways, namely the mitogen-activated protein kinase (MAPK) [36,37], Janus kinase/signal transducers and activators of transcription (JAK/STAT3) [37,38], and phosphatidylinositol-3-Kinase (PI3K)/AKT [39,40]. Notably, the PI3K/AKT pathway plays a central role in promoting cellular survival through the inhibition of apoptosis [41,42]. AKT is activated in response to doxorubicin treatment, serving as a cell survival mechanism to antagonise the antitumor effects of doxorubicin [27,43,44]. Furthermore, increased AKT activity is also implicated in chemoresistance [42,44,45]. As TFF3 promotes AKT activation [46,47], TFF3 may be a potential target to overcome acquired resistance to doxorubicin and doxorubicin associated toxicity. In this study, we showed that TFF3 reduces sensitivity and promotes acquired resistance to doxorubicin in ER+MC. The use of AMPC ((2-amino-4-(4-(6-fluoro-5-methylpyridin-3-yl)phenyl)-5-oxo-4H,5H-pyrano[3,2-c]chromene-3-carbonitrile), a novel small molecule TFF3 inhibitor, enhanced the efficacy of doxorubicin and re-sensitized the doxorubicin resistant ER+MC to doxorubicin. Therefore, TFF3 inhibition may be a promising therapeutic strategy to overcome the challenges of dose associated doxorubicin toxicity and acquired resistance in ER+MC.

## 2. Results

### 2.1. TFF3 Regulates ER+MC Cell Sensitivity to Doxorubicin

A potential correlation between doxorubicin sensitivity and TFF3 expression in a panel of ER + MC cell lines was determined using the Genomics of Drug Sensitivity in Cancer Project and Cancer Cell Line Encyclopedia (CCLE). A positive correlation (Rho = 0.643, *P*-value = 0.028) was observed between the gene expression of TFF3 and the IC_50_ of doxorubicin, suggestive that cell lines with higher TFF3 expression exhibited a lower sensitivity to doxorubicin (Figure S1). 

**Figure 1:**
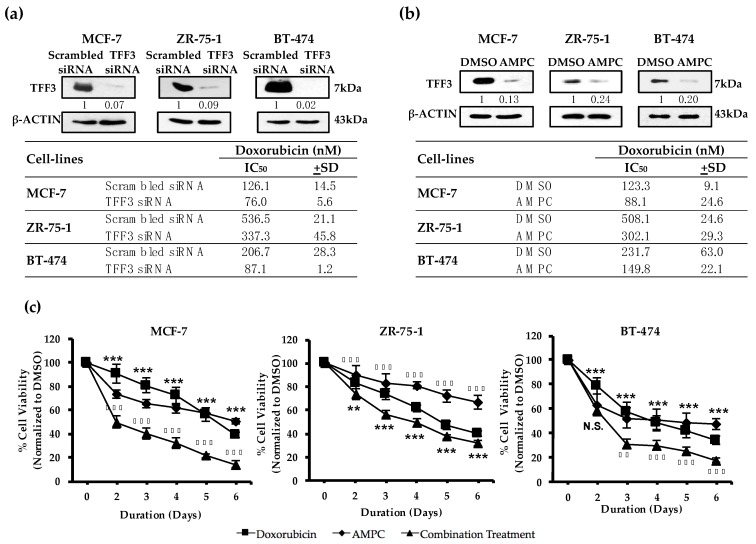

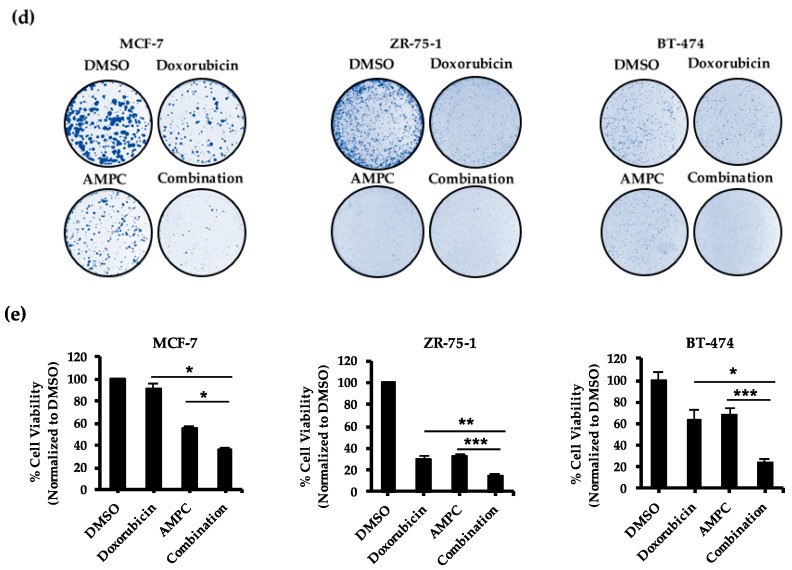
Inhibition of TFF3 enhances cellular sensitivity to doxorubicin. (**a**–**b**). MCF-7, ZR-75-1 and BT-474 cells (**a**) pre-incubated with 20nM of two TFF3 siRNA combined overnight or (**b**) treated with 10 μM AMPC were treated with increasing doses of doxorubicin for 72 h in monolayer culture. Cell viability was quantified using the AlamarBlue cell viability assay where the 50% inhibitory concentration (IC_50_) values for doxorubicin were determined using GraphPad Prism 5. Western blot analysis determined the protein expression of TFF3. β-ACTIN was used as input control. Band intensities were quantified by ImageJ and normalized to input control, where intensity ratio of scrambled siRNA/vehicle DMSO treatment was set to 1. (**c**–**e**). MCF-7, ZR-75-1 and BT-474 cells were treated with doxorubicin with or without AMPC (**c**) over a period of 6 days in monolayer culture (MCF-7: 50 nM dox, 1 μM AMPC; ZR-75-1: 200 nM dox, 5 μM AMPC; BT-474: 100 nM dox, 5 μM AMPC); (**d**) in monolayer culture at low cell density until foci formation (MCF-7: 50 nM dox, 1 μM AMPC; ZR-75-1: 25 nM dox, 2 μM AMPC; BT-474: 10 nM dox, 1 μM AMPC); and (**e**) for 9 days after 5 days pre-culture in medium containing 5% FBS and 4% Matrigel (MCF-7: 50 nM dox, 2 μM AMPC; ZR-75-1: 200 nM dox, 5 μM AMPC; BT-474: 50 nM dox, 5 μM AMPC). Cell viability was quantified using the AlamarBlue cell viability assay. Bar charts show means ± standard deviations. * denotes doxorubicin vs combination treatment; ◆ denotes AMPC vs combination treatment where * *P* < 0.05, ** *P* < 0.01 and *** *P* < 0.001 (Student’s *t*-test).

Consistently, siRNA mediated depletion of TFF3 in MCF-7 cells, using two independent TFF3 siRNAs, resulted in the enhancement of cellular sensitivity towards doxorubicin in monolayer culture. A 1.4-fold and 1.3-fold reduction in IC_50_ of doxorubicin (determined by cell viability) from 126.6 ± 1.6 nM to 89.6 ± 5.2 nM and 98.8 ± 2.5 nM was observed upon treatment with TFF3 siRNA (s277470) and (s14041) respectively (Figure S2). The combined treatment of both TFF3 siRNAs (s277470 and s14041) resulted in a greater reduction of 1.7-fold in doxorubicin IC_50_ from 126.05 ± 14.5 nM to 76.0 ± 5.6 nM in MCF-7 cells (Figure 1a). Hence, depletion of TFF3 with a combination of both TFF3 siRNAs was more effective than that of an individual TFF3 siRNA and as such, both TFF3 siRNAs were employed in combination for subsequent TFF3 depletion experiments. Consistent with that observed in MCF-7 cells, the enhanced monolayer cell sensitivity towards doxorubicin upon TFF3 depletion was also observed in two other ER+MC cell lines, ZR-75-1 and BT-474. A 1.6-fold reduction in IC_50_ of doxorubicin from 536.5 ± 21.1 nM to 337.3 ± 45.8 nM in ZR-75-1 cells; and 2.4-fold reduction from 206.7 ± 28.3 nM to 87.06 ± 1.18 nM in BT-474 cells was observed upon TFF3 depletion (Figure 1a). Hence, TFF3 depletion enhances sensitivity to doxorubicin induced ER+MC cell death.

### 2.2. Pharmacological Inhibition of TFF3 Enhances Cellular Sensitivity to Doxorubicin

A novel small molecule TFF3 inhibitor AMPC ((2-amino-4-(4-(6-fluoro-5-methylpyridin-3-yl)phenyl)-5-oxo-4*H*,5*H*-pyrano[3,2-c]chromene-3-carbonitrile) has been developed to interact specifically to the cysteine-57 residue of TFF3 required for homodimerization [12]. Consequently, AMPC disrupts homodimeric TFF3 thereby inhibiting its homodimeric functions (monomeric TFF3 functions as an antagonist to homodimeric TFF3) and promoting depletion of TFF3 (monomeric TFF3 is degraded more rapidly than dimeric TFF3) [48]. Herein, the effect of AMPC-mediated TFF3 inhibition on doxorubicin sensitivity in ER+MC was examined. As expected, AMPC significantly reduced the expression of TFF3 protein in the three ER+MC cell lines. The IC_50_ of doxorubicin (determined by cell viability) in these three cell lines grown as a monolayer was also reduced by treatment with AMPC. A 1.4 fold reduction in the IC_50_ of doxorubicin from 123.3 ± 9.1nM to 88.1 ± 24.6 nM in MCF-7; 1.7 fold reduction from 508.1 ± 24.6 nM to 302.1 ± 29.3 nM in ZR-75-1; and 1.5 fold reduction from 231.7 ± 63.0 nM to 149.8 ± 22.1 nM in BT-474 was observed upon AMPC treatment (Figure 1b). 

We next examined the cellular response to the combined treatment of AMPC with doxorubicin under monolayer, foci formation and three-dimensional Matrigel culture. The reduction in monolayer cell viability for cells treated with the combination of AMPC and doxorubicin significantly surpassed that of the individual treatments from day 2 onwards for MCF-7 and ZR-75-1, and day 3 onwards for BT-474 cells (Figure 1c). The foci formation assay is a widely used in vitro assay for determining tumour drug responses [49]. Similarly, the combined treatment of doxorubicin with AMPC exhibited a greater reduction of foci formation as compared to the individual treatments in all three ER+MC cell lines (Figure 1d). Three-dimensional Matrigel culture mimics in vivo conditions to provide an improved prediction of in vivo drug responses [50]. Consistent with the above observations, the combination treatment of AMPC and doxorubicin was also more effective in reducing 3D cell viability (Figure 1e) as compared to the individual drug treatments in all three ER+MC cell lines. This was further supported by the Live/Dead cell imaging in 3D cell culture where a greater population of dead cells was also observed in the combination treated cells than that of the individual drug treated cells (Figure S3). Hence, AMPC in combination with doxorubicin produced an improved response in ER+MC cells compared to doxorubicin alone. 

**Figure 2:**
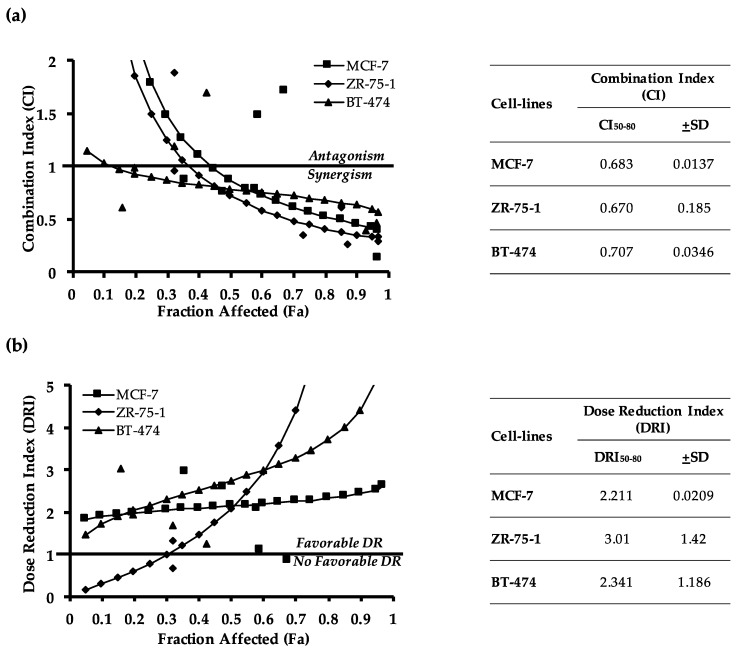
AMPC exhibits synergistic combination with doxorubicin and promotes effective dose reduction of doxorubicin. (**a**–**b**). MCF-7, ZR-75-1 and BT-474 cells were treated with increasing doses of doxorubicin and AMPC at a fixed ratio based on the IC_50_ values of the individual drugs for 72 h in monolayer culture (MCF-7 1:20, ZR-75-1 1:20, BT-474 1:25). (**a**) Combination index (CI) and (**b**) dose reduction index (DRI) were tabulated using CalcuSyn software by Chou-Talalay. CI_50-80_ and DRI_50–80_ denotes average combination index and average dose reduction index respectively at 50–80% cell death. CI > 1 indicates antagonism; CI = 1 indicates an additive effect; and CI < 1 indicates synergism. DRI > 1 is favorable dose reduction that leads to toxicity reduction.

To analyze the significance of the interactions between AMPC and doxorubicin, the combination indices of these drugs in the three ER+MC cell lines were determined. The drug combination index (CI) by Chou-Talalay is a widely used method for determining additive (CI = 1), synergistic (CI < 1), and antagonistic (CI > 1) effects of drug combinations [51]. As shown in Figure 2a, a synergistic interaction between AMPC and doxorubicin was observed in all three cell lines with the average combination index at 50–80% cell death (CI_50–80_) of 0.683 ± 0.0137, 0.670 ± 0.185 and 0.707 ± 0.0346 in MCF-7, ZR-75-1 and BT-474 cells respectively. In addition, the dose reduction index (DRI) of doxorubicin upon AMPC co-treatment was also calculated. The DRI represents the fold difference of effective dose reduction of each drug when used in combination where DRI > 1 indicates a favourable dose reduction that leads to toxicity reduction. Consistent across the three cell lines, AMPC promoted an effective dose reduction of doxorubicin when used in combination, with an average dose reduction index at 50–80% cell death (DRI_50–80_) of 2.211 ± 0.0209, 3.01 ± 1.42 and 2.341 ± 1.186 in MCF-7, ZR-75-1 and BT-474 cells respectively (Figure 2b). Hence, AMPC improved the efficacy of doxorubicin induced cell death in ER+MC cell lines.

### 2.3. AMPC Inhibits Doxorubicin-Induced AKT Activation and Enhances Doxorubicin-Induced Apoptosis

**Figure 3:**
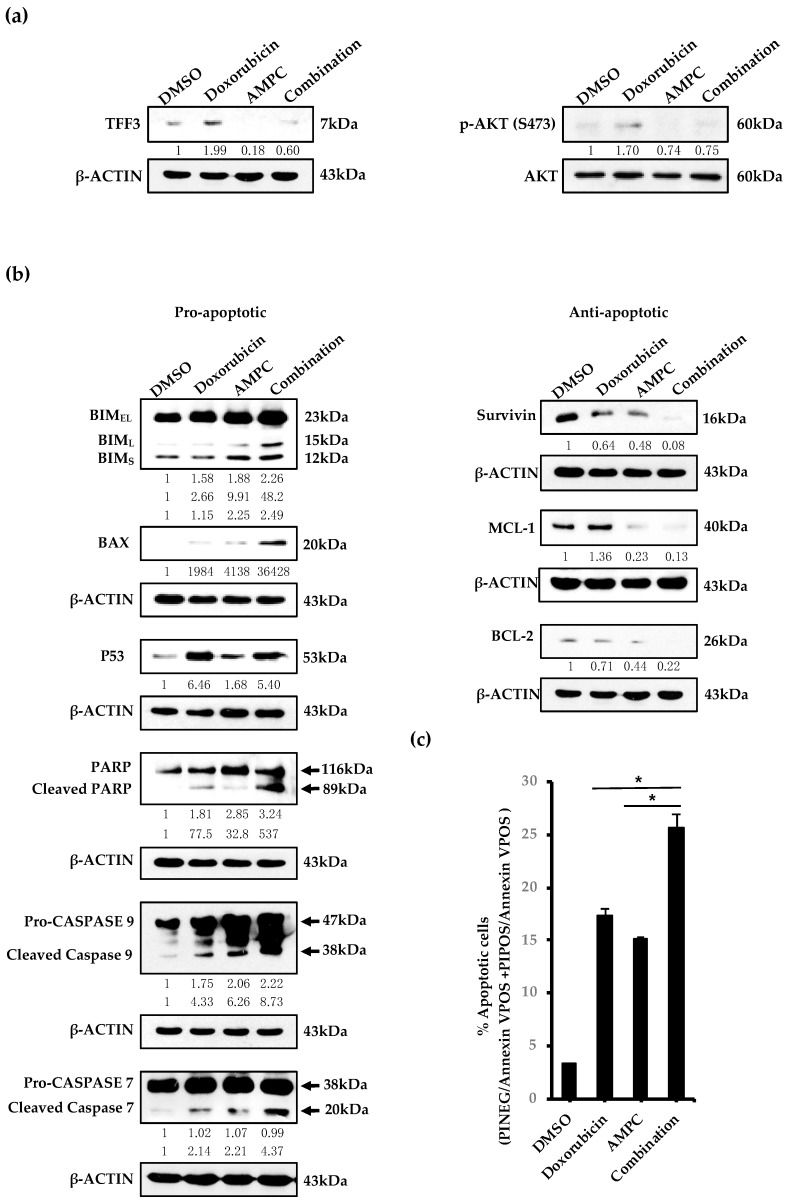
AMPC inhibits doxorubicin-induced AKT activation and enhances doxorubicin-induced apoptosis in ER+MC cells. MCF-7 cells were treated with 500nM doxorubicin with or without 10 μM AMPC in monolayer culture. (**a**–**b**). Western blot analysis determined the protein expression of (**a**) TFF3, AKT and (**b**) apoptotic-related proteins after drug treatment for 24 h. β-ACTIN was used as input control. Band intensities were quantified by ImageJ and normalized to input control/total protein for phosphorylated proteins, where intensity ratio of vehicle DMSO treatment was set to 1. (**c**) MCF-7 cells were stained with Annexin V-PI stain after drug treatment for 48 h. Percentage (%) apoptotic cells were analyzed by flow cytometry. Bar charts show means ± standard deviations. * *P* < 0.05, ** *P* < 0.01 and *** *P* < 0.001 (Student’s *t*-test).

The induction of apoptosis has been regarded as a mechanism of doxorubicin induced cell death [52,53]. Herein, doxorubicin treatment was shown to promote the cleavage of pro-apoptotic PARP, initiator caspase 9 and effector caspase 7 in ER+MC cells (Figure 3b), resulting in a total apoptotic response of 17.3% (Figure 3c). However, doxorubicin also induced the activation of the serine/threonine protein kinase AKT in ER+MC cells (Figure 3a and S4), a cell survival mechanism reported to potentially attenuate the effects of doxorubicin-induced apoptosis [27,43], Interestingly, the treatment of ER+MC cells with doxorubicin also resulted in a dose-dependent increase of TFF3 protein (Figure S4). TFF3 has previously been reported to activate AKT in hepatocellular carcinoma cells [23]. We therefore demonstrated by the use of siRNA mediated depletion of TFF3 or AMPC treatment that TFF3 depletion/inhibition decreased the activity of AKT in ER+MC cells (Figure S5a,b). Following AMPC treatment of ER+MC cells, increased pro-apoptotic protein expression including BIM, BAX and p53 as well as a decreased anti-apoptotic protein expression such as Survivin, MCL-1 and BCL-2 was observed (Figure 3b). Consequently, AMPC treatment promoted enhanced cleavage of PARP, initiator caspase 9 and effector caspase 7 (Figure 3b) in ER+MC cells, resulting in a 15.2% induction in total apoptosis (Figure 3c). 

Therefore, the effects of AMPC inhibition of TFF3 on doxorubicin-induced AKT activation and apoptosis in ER+MC cells were subsequently investigated. The combination of AMPC with doxorubicin treatment suppressed both the doxorubicin-induced TFF3 expression as well as doxorubicin-induced AKT activation in ER+MC cells (Figure 3a). Consistent with reduced AKT activation, combined treatment of AMPC with doxorubicin also promoted an increased expression of AKT-repressed pro-apoptotic proteins, BIM and BAX as compared to the single treatments in ER+MC cells (Figure 3b). In contrast, a reduction in the expression of AKT-induced anti-apoptotic protein such as Survivin, MCL-1 and BCL-2 was observed as compared to the single treatments in ER+MC cells (Figure 3b). Notably, the expression of anti-apoptotic MCL-1 and BCL-2 proteins, which were not significantly changed upon doxorubicin treatment alone, were decreased upon the combination treatment (Figure 3b). As a result, a concordant increase in total apoptosis in ER+MC was observed from 17.3% and 15.2% in doxorubicin and AMPC single treatments respectively, to 25.6% with the combination treatment (Figure 3c). The increased apoptosis observed with combination treatment was supported by the enhanced cleavage of PARP, initiator caspase 9 and effector caspase 7 in ER+MC cells upon combination treatment when compared to treatment with either doxorubicin or AMPC alone (Figure 3b). Hence, AMPC reduced the enhanced expression of TFF3 induced by doxorubicin, with consequent inhibition of the downstream doxorubicin-induced AKT activation.

### 2.4. TFF3 Expression Is Elevated in Doxorubicin Resistant ER+MC Cells 

**Figure 4:**
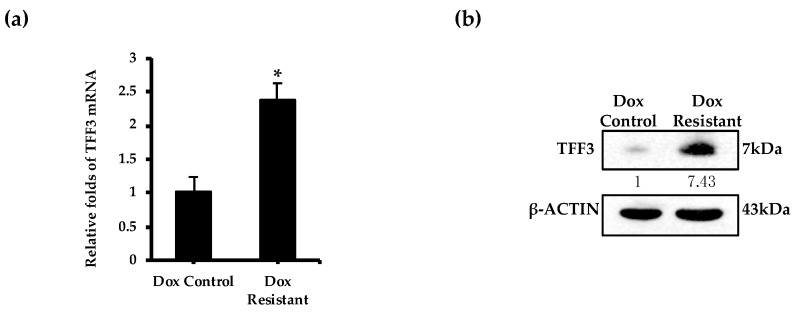
Doxorubicin resistant ER+MC cells exhibit elevated levels of TFF3. (**a**–**b**) Baseline levels of TFF3 (**a**) mRNA and (**b**) protein levels were analyzed by qPCR and western blot analysis respectively in control and doxorubicin resistant MCF-7 cells. Band intensities were quantified by ImageJ and normalized to input control, where the intensity ratio of doxorubicin control cells was set to 1. Bar charts show means ± standard deviations. * *P* < 0.05, ** *P* < 0.01 and *** *P* < 0.001 (Student’s t-test).

**Figure 5:**
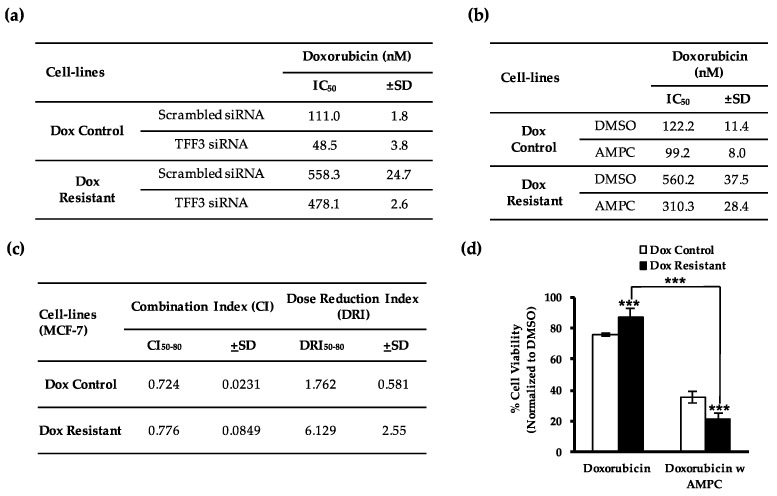

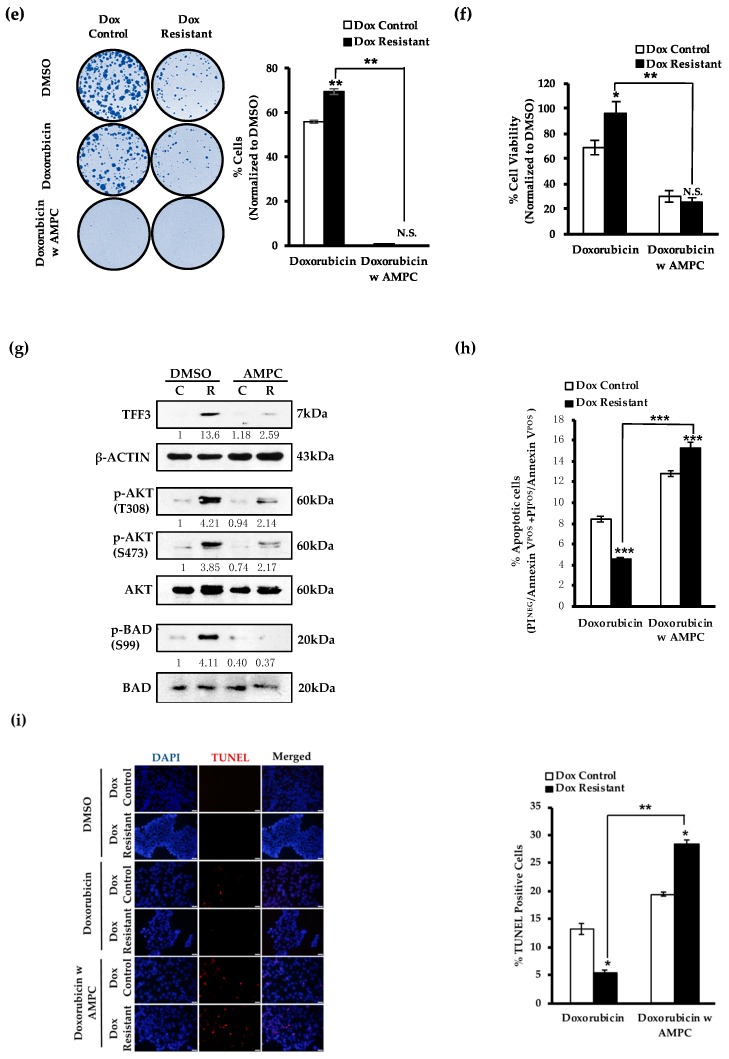
TFF3 inhibition re-sensitizes doxorubicin resistant ER+MC cells to doxorubicin-induced apoptosis. Control and doxorubicin resistant MCF-7 cells (**a**) pre-incubated with 20 nM of two TFF3 siRNA combined or (**b**) co-treated with 1 μM AMPC were treated with increasing doses of doxorubicin for 72 h in monolayer culture. (**c**) Increasing doses of doxorubicin and AMPC were treated to control and doxorubicin resistant MCF-7 cells at a fixed ratio of 1:20 for 72 h in monolayer culture. Combination index (CI) and dose reduction index (DRI) were tabulated using CalcuSyn software by Chou-Talalay [51]. CI_50–80_ and DRI_50–80_ denotes average combination index and average dose reduction index respectively at 50–80% cell death. CI > 1 indicates antagonism; CI = 1 indicates an additive effect; and CI < 1 indicates synergism. DRI > 1 is favorable dose reduction that leads to toxicity reduction. (**d**–**f**). Control and doxorubicin resistant ER+MC cells were treated with doxorubicin with or without AMPC (**d**) for 3 days in monolayer culture (50 nM dox, 1 μM AMPC); (**e**) in monolayer culture at low cell density until foci formation (25 nM dox, 2 μM AMPC); and (**f**) for 9 days after 5 days pre-culture in medium containing 5% FBS and 4% Matrigel (100 nM dox, 5 μM AMPC). (**g**) Western blot analysis for protein levels of TFF3, AKT and BAD after 10 μM AMPC treatment for 24 h. C denotes control cells while R denotes doxorubicin resistant ER+MC cells. β-ACTIN was used as input control. Band intensities were quantified by ImageJ and normalized to input control/total proteins for phosphorylated proteins, where intensity ratio of control cells treated with vehicle DMSO was set to 1. (**h**–**i**). Total apoptosis was analyzed in the control and doxorubicin resistant MCF-7 cells treated with combination of doxorubicin with or without AMPC for (**h**) 48 h, followed by Annexin V-PI staining and quantification by flow cytometry; or (**i**) 24 h, followed by TUNEL staining and visualization by fluorescent microscopy. % of TUNEL positive cells was quantified by ImageJ. Cell viability was quantified using the AlamarBlue cell viability assay and 50% inhibitory concentration (IC_50_) values for doxorubicin were determined using GraphPad Prism 5. The scale bar represents 50 μm. Bar charts show means ± standard deviations. * *P* < 0.05, ** *P* < 0.01 and *** *P* < 0.001 (Student’s *t*-test).

To determine a potential role of TFF3 in acquired doxorubicin resistance in ER+MC, doxorubicin resistant ER+MC cells were generated and characterized (Figure S6). Based on the IC_50_ of doxorubicin, the doxorubicin resistant ER+MC cells generated were approximately four times more resistant than that of the control cells with a monolayer IC_50_ of doxorubicin (determined by cell viability) of 561.0 ± 52.47 nM and 137.45 ± 37.12 nM respectively (Figure S6a). Importantly, elevated levels of TFF3 mRNA and protein were observed in doxorubicin resistant ER+MC cells as compared to the control cells (Figure 4a,b). siRNA-mediated depletion of TFF3 in the doxorubicin resistant ER+MC cells produced a partial re-sensitization to doxorubicin treatment observed by a decrease in the monolayer IC_50_ of doxorubicin (determined by cell viability) from 558.3 ± 24.7 nM to 478.1 ± 2.6 nM (Figure 5a). 

### 2.5. AMPC Partially Re-Sensitizes Doxorubicin Resistant ER+MC Cells to Doxorubicin 

We next determined the potential of AMPC to re-sensitize doxorubicin resistant ER+MC cells to doxorubicin. Consistent with siRNA-mediated depletion of TFF3, the inhibition of TFF3 by AMPC also partially resensitized doxorubicin resistant ER+MC cells to doxorubicin, resulting in a decrease in the monolayer IC_50_ of doxorubicin (determined by cell viability) from 560.2 ± 37.5 nM to 310.3 ± 28.4 nM (Figure 5b). Despite only partially reducing the IC_50_, the combination of AMPC with doxorubicin significantly reduced the overall monolayer cell viability of the resistant cells from 87.5% ± 5.4% to 21.8% ± 3.7% as compared to doxorubicin only (Figure 5d). Such reductions were similarly observed with foci formation and growth in 3D conditions where a 69.2% ± 1.2% and 72.0% ± 7.7% further reduction in cell viability was observed respectively when AMPC was combined with doxorubicin treatment (Figure 5e and f). Moreover, a synergistic interaction between AMPC and doxorubicin (CI_50–80_ of 0.776 ± 0.0849) was also observed in the doxorubicin resistant ER+MC cells where the addition of AMPC greatly reduced the required dose of doxorubicin (DRI_50–80_ = 6.129 ± 2.55) (Figure 5c).

In addition to increased TFF3 expression, the doxorubicin resistant ER+MC cells exhibited increased AKT activation with consequent increased downstream BAD phosphorylation on Ser99 (Figure S6c). The resistant cells also exhibited reduced expression of pro-apoptotic proteins including BIM, BAX, P53 and PUMA and increased expression of anti-apoptotic proteins such as Survivin, MCL-1, BCL-2 and BCL-xL (Figure S6c). Consequently, the doxorubicin resistant ER+MC cells were more resistant to doxorubicin-induced apoptosis than the control cells as shown in the TUNEL and Annexin V/PI apoptosis assays (Figure S6d,e). The treatment of doxorubicin resistant ER+MC cells with AMPC suppressed both the elevated expression of TFF3 as well as the enhanced AKT activation observed in the resistant cells (Figure 5g). The enhanced BAD phosphorylation was also inhibited, with reduced BAD phosphorylation observed in the resistant cells upon AMPC treatment (Figure 5g). As such, re-sensitization of the doxorubicin resistant ER+MC cells to doxorubicin-induced apoptosis was observed, from 4.6% when treated with only doxorubicin to 15.4% total apoptosis when AMPC was combined with doxorubicin (Figure 5h). This was further supported by the TUNEL assay, showing an increased apoptosis-associated DNA fragmentation in the doxorubicin resistant ER+MC cells treated with a combination of AMPC and doxorubicin as compared to the doxorubicin alone treated cells (Figure 5i). Hence, the inhibition of TFF3 by AMPC suppressed the increased AKT activity in the doxorubicin resistant ER+MC cells, resulting in the re-sensitization of the resistant cells to doxorubicin-induced apoptosis. 

### 2.6. Potential Use of AMPC as a Monotherapy in Doxorubicin Resistant ER+MC

**Figure 6:**
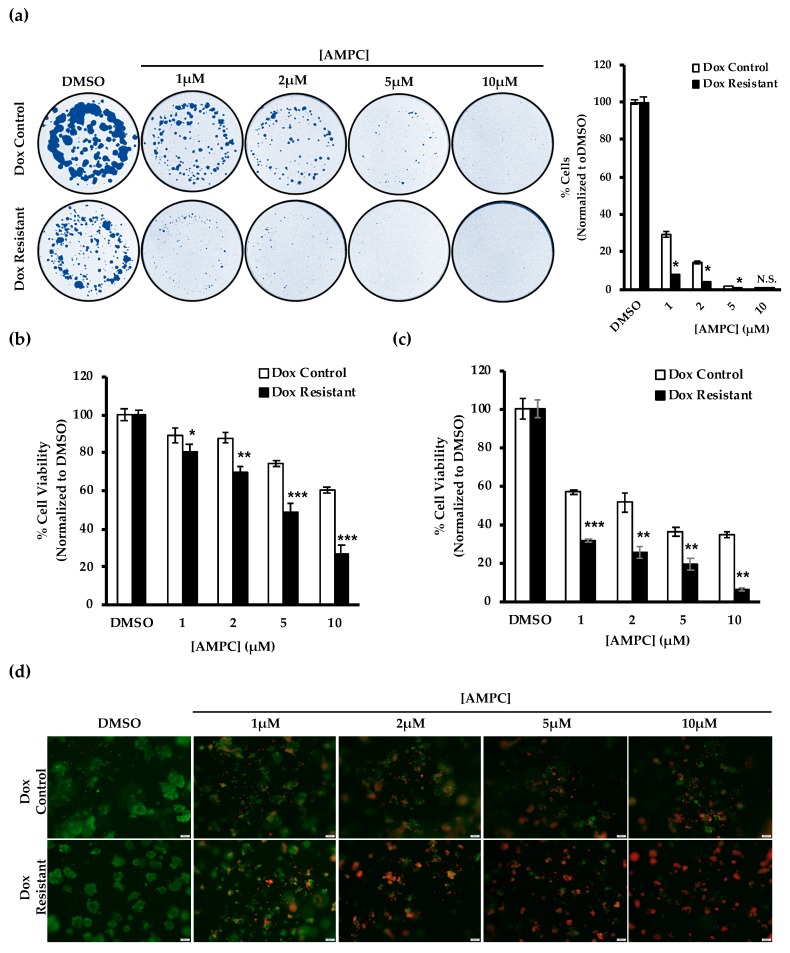
Doxorubicin resistant ER+MC cells exhibit enhanced sensitivity to AMPC. Control and doxorubicin resistant ER+MC cells were treated with indicated doses of AMPC (**a**) in monolayer culture at low cell density until foci formation; (**b**) for 3 days in monolayer culture; (**c**–**d**) for 9 days after 5 days pre-culture in medium containing 5% FBS and 4% Matrigel and subsequently stained with LIVE/DEAD cell viability assay and visualized using florescence microscopy. Red indicates dead cells and green indicates live cells. Cell viability was quantified using the AlamarBlue cell viability assay. Staining intensity of foci formation was quantified by ImageJ. The scale bar represents 200μm. Bar charts show means ± standard deviations. * *P* < 0.05, ** *P* < 0.01 and *** *P* < 0.001 (Student’s t-test).

The above results suggested that doxorubicin resistant ER+MC cells exhibit a greater sensitivity to AMPC treatment as compared to the control cells. To explore the potential use of AMPC as a monotherapy in doxorubicin resistant ER+MC, doxorubicin resistant ER+MC cells were exposed to different doses of AMPC *in vitro*. Indeed, the doxorubicin resistant ER+MC cells were more sensitive to AMPC than that of the control sensitive cells (Figure 6). 

Single treatment with AMPC resulted in a greater percentage reduction of monolayer viability, foci formation and 3D cell viability in the doxorubicin resistant ER+MC cells as compared to the control cells in a dose-dependent manner (Figure 6a,b,c). For example, when examining ER+MC cells pre-grown in 3D Matrigel, treatment with 10 μM AMPC reduced doxorubicin resistant ER+MC cell viability to 6% whereas the control ER+MC cell viability was reduced to approximately 34% (Figure 6c). The Live/Dead cell imaging in 3D cell culture also revealed a greater population of dead cells in the doxorubicin resistant ER+MC cells than that of the control cells across the different doses of AMPC treated (Figure 6d). Hence, the elevated TFF3 expression and greater sensitivity to AMPC in the doxorubicin resistant ER+MC cells suggest that the resistant cells may exhibit a greater reliance on TFF3 for survival than the control cells.

### 2.7. Inhibition of TFF3 Produces Regression of Doxorubicin Resistant ER+MC xenografts

**Figure 7:**
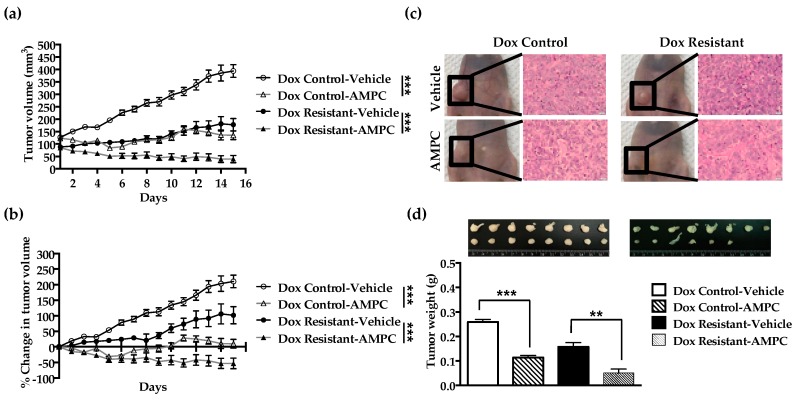

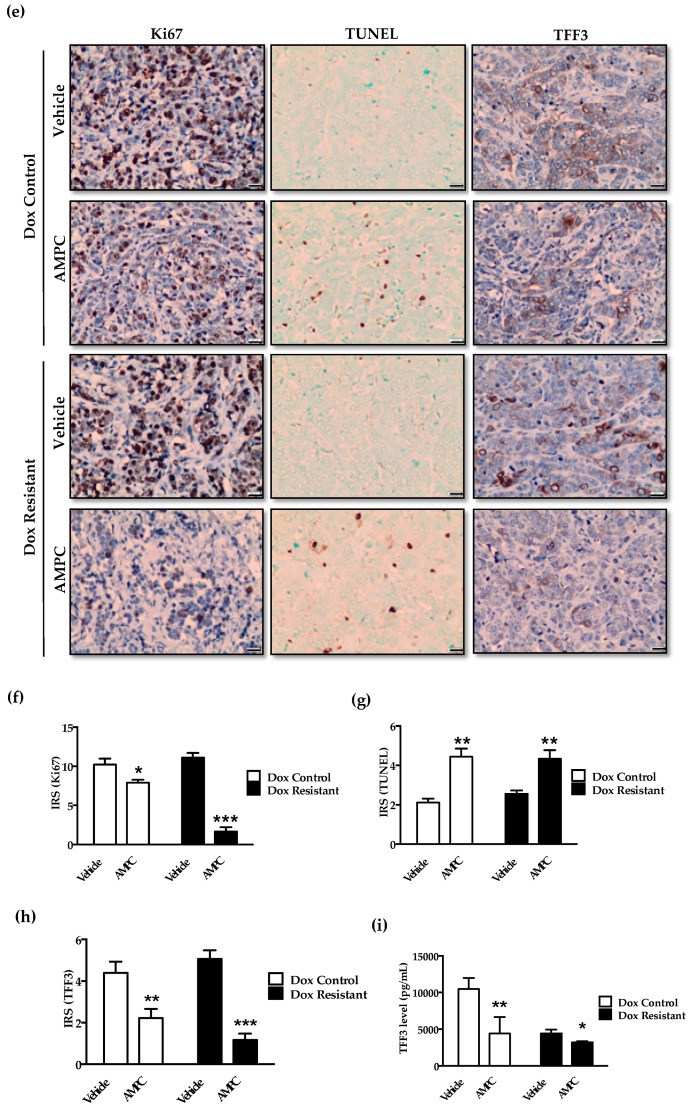
Inhibition of TFF3 by AMPC inhibits the in vivo growth of ER+ MC xenografts. Doxorubicin control and resistant ER+ MCF-7 cells were injected orthotopically into the mammary fat pad of nude mice. (**a**) Tumour volumes and (**b**) % change in tumour volume in mice were recorded daily with vehicle or AMPC treatment over 15 days. (**c**) Representative photographs of tumour sites (left) and the corresponding micrographs of the H&E-stained tumour sections at 200× magnification (right). (**d**) Representative photographs of tumours from each treatment groups and the average tumour weights. (**e**–**h**) Immunohistochemistry (IHC) staining was performed on tumours for Ki67, TUNEL and TFF3. Representative micrographs were taken at 200x magnification and staining intensity were scored using the immunoreactivity scoring (IRS) system. (**i**) Serum levels of TFF3 was determined by ELISA. Bar charts represents the mean ± SD of the averages of 6-8 mice scored. Scale bars represents 20 μm. Results were statistically analysed with one-way ANOVA followed by Tukey’s post hoc test. Statistical significance, * P < 0.05, ** P < 0.01 and *** P < 0.001, compared with the vehicle-treated group.

To further examine the efficacy of AMPC used as monotherapy, orthotopic mouse xenograft models generated with the doxorubicin control and resistant ER+MC cells were treated with AMPC. AMPC significantly reduces the average tumour volume and weight of both the control and doxorubicin resistant ER+MC xenografts (Figure 7a–d). Consistent with the in vitro findings, doxorubicin resistant ER+MC xenografts also exhibited a greater in vivo sensitivity to AMPC monotherapy than that of the doxorubicin control xenografts.

A greater reduction in average tumour volume of approximately 4.5 fold from 178 mm^3^ to 40 mm^3^ was observed in the doxorubicin resistant ER+MC xenografts at day 15 as compared to a 2.9 fold reduction from 394 mm^3^ to 135 mm^3^ in the doxorubicin sensitive xenografts, upon AMPC treatment (Figure 7a). Similarly, AMPC also produced a greater reduction in the average tumour weight of the doxorubicin resistant ER+MC xenografts from 0.15 g to 0.05 g (approximately 3-fold) as compared to the doxorubicin control xenografts from 0.26 g to 0.11 g (approximately 2.4-fold) (Figure 7d). Indeed, AMPC treatment resulted in the complete regression of two of the doxorubicin resistant tumours as shown in Figure 7d. Reduction of serum TFF3 levels and tumour expression of TFF3 by AMPC was accompanied by decreased expression of the proliferative marker Ki67 in both the doxorubicin sensitive and resistant ER+MC xenografts (Figure 7e,f,h,i). AMPC treatment produced an increased apoptosis-associated DNA fragmentation as indicated by TUNEL staining (Figure 7g). At the dose and schedule used herein, AMPC treatment appeared well tolerated by animals with no significant changes in body weight. Consistently, the appearance and weight of vital organs of the AMPC-treated mice including liver, spleen and kidney were not different compared to those of vehicle-treated mice (Figure S8). Hence, this data indicates the potential utility of AMPC as a single agent for the treatment of doxorubicin resistant ER+MC. 

## 3. Discussion

Herein, we have demonstrated that TFF3 decreases doxorubicin sensitivity and mediates acquired doxorubicin resistance in ER+MC. Consistently, the use of AMPC, a novel small molecule TFF3 inhibitor enhanced ER+MC cell sensitivity to doxorubicin and also overcame acquired doxorubicin resistance. In support of these findings, literature evidence has also reported that TFF3 reduces doxorubicin sensitivity in gastric [33] and liver cancer [23]. In addition to TFF3 reduction of doxorubicin sensitivity, TFF3 has been reported as a biomarker of poor response to various chemotherapeutic agents [31]. A recent study has demonstrated that high TFF3 expression is associated with residual disease after neoadjuvant chemotherapy of invasive ductal carcinoma whereas low TFF3 expression is associated with a complete pathological response [54]. Consistently in our study, elevated levels of TFF3 are observed in ER+MC with acquired doxorubicin resistance, whilst the depletion or AMPC-mediated inhibition of TFF3 partially re-sensitized the resistant cells to doxorubicin-induced cytotoxicity and apoptosis. Hence, we have provided functional evidence for the observed clinical association of TFF3 expression with residual disease after neoadjuvant chemotherapy. Nevertheless, we observed that TFF3 inhibition did not completely abrogate doxorubicin resistance in ER+MC. This may be attributed to the highly elevated TFF3 protein level and activity in the doxorubicin resistant ER+MC cells, which may not have been sufficiently decreased or inhibited by siRNA-mediated depletion or AMPC treatment respectively. Alternatively, the involvement of other non-TFF3 mediated mechanisms of acquired doxorubicin resistance in our cell model may also have contributed to this observation. 

The AKT survival pathway is activated as part of the cellular survival response against doxorubicin treatment [43,44]. Indeed, studies have demonstrated the effectiveness of inhibiting doxorubicin-induced AKT activity to improve the response to doxorubicin [27,55,56]. In addition, increased activation of AKT has also been associated with chemoresistance in MC, including to doxorubicin, and inhibition of AKT activity reverses the resistance [43,57,58]. Herein, increased AKT activity together with the elevated TFF3 expression were observed in response to doxorubicin treatment and in acquired doxorubicin resistant ER+MC. This is consistent with the observation in neoadjuvant chemotherapy resistant MC where TFF3 expression and phosphorylated-AKT1 were co-localized [54]. Considering the pro-survival properties of TFF3 [12,13,14,15], as well as its downstream activation of AKT [39,40,46,59], it can be concluded that TFF3 promotes the activation of AKT leading to the inhibition of doxorubicin-induced apoptosis. This results in the reduction of doxorubicin sensitivity as well as the promotion of doxorubicin resistance. In agreement, the pharmacological inhibition of TFF3 by AMPC was shown to suppress doxorubicin-induced AKT activation in wild-type ER+MC cells, as well as the increased activation of AKT in the doxorubicin resistant ER+MC cells. As a result, an enhanced doxorubicin-induced apoptosis was observed following the inhibition of TFF3 by AMPC in both the wild-type and doxorubicin resistant ER+MC cells. Hence, this data provides a molecular mechanism for TFF3 regulation of doxorubicin sensitivity and resistance in ER+MC.

The direct inhibition of AKT has been shown to result in the reactivation of various receptor tyrosine kinases (RTKs) associated with cellular survival such as HER2, HER3, HER4 and IGF-1R, in a negative feedback loop [60,61,62]. This is one major limitation hindering the effective use of AKT inhibitors as single agents [60,61]. In contrast, TFF3 behaves as a promiscuous activator of various survival pathways [37,38,47]. In addition to reducing downstream AKT activation, the inhibition of TFF3 has also been shown to inhibit the activation of other cell survival associated RTKs such as epidermal growth factor receptor (EGFR), HER2, HER3, HER4, MET and IGF-1R [32]. Given the limitations of inhibiting AKT alone, the combinatorial inhibition of AKT and RTKs has been deemed as a more effective strategy in inhibiting cancer cell survival [62,63]. Promising efficacy of the combination of MK-2206 (AKT inhibitor) with lapatinib (HER1/2 inhibitor) in advanced HER2+ MC [64], as well as MK-2206 with selumetinib (MEK1/2 inhibitor) [65] in advanced or metastatic solid tumours have also been reported in phase I clinical trials. This highlights a potential superiority of inhibiting TFF3 over AKT inhibition. Hence, the inclusion of a TFF3 inhibitor in doxorubicin-containing regimens for ER+MC patients would enhance the efficacy of doxorubicin, thereby reducing its dose requirement and the associated dose-dependent toxicity. In contrast, the markedly higher expression of TFF3 (and increased AKT activation) in doxorubicin resistant ER+MC cells, as compared to control cells, may have contributed to the incomplete abrogation of doxorubicin resistance by AMPC. Therefore, a dual inhibition of both AKT and TFF3 may be a more effective approach to abrogate doxorubicin resistance in ER+MC.

Although the inhibition of TFF3 did not completely abrogate doxorubicin resistance in ER+MC cells, we observed that these resistant cells were more susceptible to TFF3 inhibition than the control cells. As observed, a greater reduction in both the in vitro cell viability and in vivo tumour burden was evident in doxorubicin resistant ER+MC as compared to doxorubicin sensitive ER+MC following the inhibition of TFF3 by AMPC. This suggests the effectiveness of TFF3 inhibition as a monotherapy to eliminate doxorubicin resistant ER+MC cells despite only partial re-sensitization to doxorubicin. This further suggests that the enhanced sensitivity of doxorubicin resistant ER+MC cells to TFF3 inhibition may be due to the addiction of these cells to TFF3 for their survival. As such, TFF3 expression may also be a potential predictive biomarker of response to TFF3 inhibition in doxorubicin resistant ER+MC. 

## 4. Materials and Methods 

### 4.1. Cell Culture and Transfection

The human ER+MC cell lines, MCF-7, ZR-75-1 and BT474 were obtained from American Type Culture Collection (ATCC, Rockville, MD, USA) and cultured under ATCC recommended conditions at 37 °C with 5% CO_2_. All cell lines were cultured in complete RPMI 1640 medium (Gibco, Carlsbad, CA, USA) supplemented with 10% Fetal Bovine Serum (FBS) (Biowest, Riverside, MO, USA) and 1% Penicillin Streptomycin (PS) (Invitrogen, Carlsbad, CA, USA). 

Cells cultured at 50–70% confluency were transiently transfected with TFF3 siRNA, or scrambled siRNA as negative control, using Lipofectamine 2000 (Life Technologies, Carlsbad, CA, USA) according to the manufacturer’s instructions. The siRNA oligonucleotides used were namely Silencer^®^ Select Negative Control No. 1 siRNA and Silencer^®^ Select TFF3 siRNA (s277470 and s14041) obtained from Thermo Fisher Scientific (Waltham, MA, USA). 

### 4.2. Generation of Doxorubicin Resistant Cell Lines

Doxorubicin was purchased from Sigma-Aldrich (St. Louis, MO, USA) and dissolved in dimethyl sulfoxide (DMSO) at a stock concentration of 30 mM. To generate the acquired doxorubicin resistant cell line, MCF-7 cells seeded at 30–50% confluency were exposed to 6 pulses of doxorubicin (Sigma-Aldrich, St. Louis, MO, USA) at its half maximal inhibitory concentration (IC_50_) in complete RPMI supplemented with 10% FBS and 1% PS for 24 h each followed by a recovery period of one week at 37 °C with 5% CO_2_. Control cells were maintained alongside in complete RPMI containing DMSO. Doxorubicin resistance was assessed by determination of doxorubicin IC_50_ wherein cells seeded at 5x10^3^ cells/well in 96-well microtiter plates were exposed to increasing concentrations of doxorubicin prepared in complete media for 72 h at 37 °C with 5% CO_2_. Cell viability was quantified by 10% AlamarBlue (Life Technologies, Carlsbad, CA, USA) according to the manufacturer’s protocol. Fluorescence was determined using a microplate reader (Infinite 200, Tecan, Mannedorf, Zurich, Switzerland) at 560nm and 590nm for excitation and emission respectively. IC_50_ values were tabulated using Graphpad Prism 5 software (GraphPad Software, Inc., San Diego, CA, USA). 

### 4.3. Cell Viability Assay

Cell viability assays were performed under monolayer and three- dimensional (3D) Matrigel culture conditions. In monolayer culture, cells were seeded at 2–5 × 10_3_ cells/well in 96-well microtiter plates and left to adhere overnight. Cells were exposed to drugs in complete RPMI for up to 6 days at 37 °C with 5% CO_2_. Cell viability was quantified by 10% AlamarBlue wherein fresh media containing drugs were replaced daily thereafter. 

In three-dimensional culture, cell suspensions were prepared in media containing 5% FBS and 4% Matrigel (BD Biosciences, San Jose, CA, USA) and subsequently seeded at a density of 1x10_3_ cells/well in 96-well microtiter plates with growth factor-reduced Matrigel. Spheroids were allowed to form for 5 days before drugs prepared in complete media were added. Fresh media containing drugs were replaced every 3 days for up to 9 days at 37°C with 5% CO_2_. Cell viability was quantified by 10% AlamarBlue. Cells were stained by LIVE/DEAD staining kit (Invitrogen, Carlsbad, CA, USA) according to manufacturer’s instruction and visualized under an IX71 fluorescence microscope (Olympus Life Science, Shinjuku, Tokyo, Japan) at 4× magnification. Images were captured using CellSens imaging software (Olympus Life Science, Shinjuku, Tokyo, Japan). 

### 4.4. Foci-Formation Assay

Cells were cultured in 6 well plates at a cell density of 1 × 10_3_ cells/well. Complete media containing drugs were added 24 h later and replaced every 3 days at 37 °C with 5% CO_2_ until foci formation. Foci formed were first fixed in ice cold methanol for 10 min and stained with 0.5% crystal violet (Sigma Aldrich, St. Louis, MO, USA) for another 10 min before visualizing using the Gel Doc XR Imaging system (Bio-Rad Laboratories, Hercules, CA, USA). Images were captured using the ImageLab software (Bio-Rad Laboratories, Hercules, CA, USA).

### 4.5. mRNA Expression Analysis

Total RNA was isolated from cells seeded at 50–70% confluency using the RNeasy mini Kit (Qiagen, Hilden, Germany) and subsequently converted into cDNA using the Superscript VILO cDNA synthesis kit (Life Technologies, Carlsbad, CA, USA) according to manufacturer’s instructions. mRNA expression levels were then quantified through real-time quantitative PCR (qPCR) as previously described (23). The primers used for qPCR are as follows: *TFF3* forward 5′-CTTGCTGTCCTCCAGCTCT-3′, *TFF3* reverse 5′- CCGGTTGTTGCACTCCTT-3′, β-*Actin* forward 5′- GCACTCTTCCAGCCTTCCTT-3′ and β-*Actin* reverse 5′- GCACTCTTCCAGCCTTCCTT-3′.

### 4.6. Protein Expression Analysis 

Cells seeded at 50–70% confluency were lysed in protein lysis buffer containing 1× protease inhibitor (Roche, Mannheim, Germany) and 1 x phosphatase inhibitor (Roche, Mannheim, Germany). Protein expression was analysed by western blot analysis as previously described (23). Individual blots were cut into multiple sections dependent on molecular weight to facilitate the detection of multiple proteins. The primary antibodies used are as follows: TFF3 (ab108599, Abcam, Cambridge, UK); β-Actin (sc-47778m, Santa Cruz Biotechnology, Inc., Santa Cruz, CA, USA); phospho-AKT1 S473 (ab66138, Abcam, Cambridge, UK); phospho-AKT1 T308 (4056S, Cell Signaling Technology, Danvers, MA, USA); pan-AKT (ab8805, Abcam, Cambridge, UK); phospho-FOXO3a S253 (13129S, Cell Signaling, Danvers, MA, USA); FOXO3a (2497S, Cell Signaling, Danvers, MA, USA); BIM (2933S, Cell Signaling, Danvers, MA, USA); Bax (5023S, Cell Signaling, Danvers, MA, USA); P53 (sc-98, Santa Cruz Biotechnology, Inc., Santa Cruz, CA, USA); Survivin (2808S, Cell Signaling, Danvers, MA, USA); MCL-1 (94296S, Cell Signaling, Danvers, MA, USA); BCL-2 (4223S, Cell Signaling, Danvers, MA, USA); PARP (9532S, Cell Signaling, Danvers, MA, USA); Caspase 9 (9508S, Cell Signaling, Danvers, MA, USA); Caspase 7 (12827S, Cell Signaling, Danvers, MA, USA); PUMA (12450S, Cell Signaling, Danvers, MA, USA); BCL-xL (2764S, Cell Signaling, Danvers, MA, USA). The secondary anti-rabbit and anti-mouse horseradish peroxidase (HRP)-conjugated antibodies used were obtained from Cell Signaling Technology. 

### 4.7. Annexin-V/ Propidium Iodide (PI) Staining 

Apoptotic cell population was quantified using the Dead Cell Apoptosis Kit with Annexin V FITC and PI (Invitrogen, Carlsbad, CA, USA). Cells were seeded at 50–70% confluency and left to adhere overnight. Culture media were then aspirated and replaced with complete media containing drugs at the desired concentration and incubated for 48 h at 3 °C with 5% CO_2_. Both adherent and floating cells were harvested, pelleted and washed in PBS before re-suspending in annexin V binding buffer. Cells were then stained with 100 μg/mL propidium iodide and 1 μL FITC annexin V for 15 min at room temperature before being assessed and analyzed by FACSAriaII flow cytometer (BD Biosciences, San Jose, CA, USA) and BD FACSDIVA software (BD Biosciences, San Jose, CA, USA), respectively. 

### 4.8. TUNEL Assay

Cells were seeded at a cell density of 2 × 10^4^ cells/well in 2-chamber slides where drugs in complete media were added for 24 h at 37 °C with 5% CO_2_. Treated cells were fixed in 4% paraformaldehyde (PFA) (Sigma- Aldrich, St. Louis, MO, USA), permeabilized by 0.1% Triton X-100 (Sigma- Aldrich, St. Louis, MO, USA) and stained with TUNEL assay kit (Roche, Mannheim, Germany) accordingly to the manufacturer’s instructions before being mounted with DAPI containing mounting media (Vector Laboratories, Burlingame, CA, USA). Slides were visualized under the Olympus IX71 fluorescence microscope at 20× magnification. Images were captured using CellSens imaging software.

### 4.9. Analysis of Combination Index (CI)

The type of drug-drug interactions between AMPC and doxorubicin was determined using the combination index (CI) by Chou and Talalay. This methodology is based on the median-effect equation, derived from the mass-action law principle [51]. Drug-drug interactions can be distinguished as additive, synergistic or antagonistic based on the CI values where CI > 1 indicates antagonism; CI = 1 indicates an additive effect; and CI < 1 indicates synergism. 

Cells were seeded at a cell density 5 × 10^3^ cells/well in 96-well microtiter plates. Complete media containing increasing doses of doxorubicin and AMPC individually or in combination were added for 72 h at 37 °C with 5% CO_2_. Doses of doxorubicin and AMPC used were kept at a fixed ratio of 1:20 based on the differences in the IC_50_ of these two drugs. Cell viability was quantified by 10% AlamarBlue where CI and dose reduction index (DRI) values were then subsequently determined using the CalcuSyn software. DRI > 1 indicates favorable dose reduction that leads to toxicity reduction. 

### 4.10. In Vivo Tumour Xenograft

Four to five week-old female BALB/c athymic nude mice were obtained from the Vital River Laboratory Animal Technology Co., Ltd. (Haidian district, Beijing, China) and acclimated for 1 week. All animals were housed in a controlled atmosphere (25 ± 1 °C at 50% relative humidity) under a 12-h light/12-h dark cycle. Animals had free access to food and water at all times. Food cups were replenished with fresh diet daily. All animal experimental protocols used in this study were approved by the Institutional Animal Care and Use Committee of the Laboratory Animal Centre of Peking University Shenzhen Graduate School (the permit from Peking University Shenzhen Graduate School is “YW”: the permit from Tsinghua Shenzhen International Graduate School is “ethical development no. 37 (Year 2019)”. All mice were implanted subcutaneously with 0.72 mg of 60-day release 17β-estradiol pellets (Innovative Research of America, Sarasota, FL, USA), and 1.5 × 10^7^ doxorubicin control and resistant ER+MC cells were injected into the right fourth mammary fat pat (with Matrigel). Eight days after injection (tumour size approximately 80–100 mm^3^), the mice were randomized into 4 groups (*n* = 8) and injected intraperitoneally with or without daily doses of AMPC at 40 mg/kg body weight for 2 weeks (summarized in Figure S7). Animal body weight and orthotopic tumour volume were monitored daily using an electronic balance and a digital square, respectively. Tumour volume (mm^3^) was calculated by using the formula: 0.52 × length × [width]^2^. All mice were sacrificed after 21 days, and the tumour masses were dissected. The dissected tumours were processed for routine histological examination.

### 4.11. Quantification of Serum TFF3 Levels

The mouse serum was collected, centrifuged and stored at −80 °C prior to testing. The TFF3 levels in the serum were quantified through colorimetric determination using a specific human TFF3 Quantikine ELISA Kit (DTFF30, R&D Systems, Minneapolis, MN, USA) according to the manufacturer’s instructions.

### 4.12. Statistical Analysis

All numerical data are expressed as mean ± standard deviation (SD) from a representative experiment performed in triplicate. Correlation analysis for the association between TFF3 expression and sensitivity to doxorubicin in a panel of MC cell lines was carried out using the Spearman’ s rho correlation coefficient. Statistical significance was assessed by an unpaired two-tailed Student’s t-test (P < 0.05 was considered as significant).

## 5. Conclusions

TFF3 reduced sensitivity and promoted acquired resistance towards doxorubicin in ER+MC by suppression of doxorubicin-induced apoptosis mediated via the AKT pathway. The pharmacological inhibition of TFF3 by the small molecule TFF3 inhibitor (AMPC) enhanced the efficacy of doxorubicin in doxorubicin sensitive ER+MC cells, with synergistic combinatorial effects. Moreover, AMPC as a single agent exhibited single agent efficacy in doxorubicin resistant ER+MC cells, and in a xenograft model, suggestive of increased dependency of doxorubicin resistant ER+ MC cells on TFF3 for cell survival. Hence, we propose the use of TFF3 inhibitors to provide effective doxorubicin dose reduction (to avoid dose-dependent toxicity) and to abrogate acquired resistance to doxorubicin in ER+ MC patients with increased TFF3 expression. 

## 6. Patents

V.P., B., K.S.R., and P.E.L. are named as inventors on PCT application SG2018/050277 (WO2018226155), (13/12/2018), Compounds, As Inhibitors of TFF3 Dimerization, Methods and Applications Thereof. 

## Supplementary Materials

The following are available online at https://www.mdpi.com/2072-6694/11/10/1528/s1, Figure S1: Spearman’s rank correlation between TFF3 mRNA expression and doxorubicin sensitivity in a panel of 12 ER+ MC cell lines, Figure S2: Combined use of two independent TFF3 siRNAs exhibit greater efficacy in enhancing cellular sensitivity towards doxorubicin, Figure S3: AMPC-mediated inhibition of TFF3 enhances doxorubicin-induced cell death, Figure S4: Doxorubicin induces TFF3 expression and AKT activation, Figure S5: Depletion/inhibition of TFF3 decreases the activation of AKT, Figure S6: Characterization of acquired doxorubicin resistant ER+MC cells, Figure S7: Study design for orthotopic mouse model, Figure S8: Changes in the body weights and appearances of vital organs of mice treated with AMPC, Figure S9: Original blots from Figure 1a and b, Figure S10: from Figure 3a and b, Figure S11: from Figure 4b, Figure S12: from Figure 5g.
