# Supplementary material for: Inhibition of TFF3 Enhances Sensitivity—and Overcomes Acquired Resistance—to Doxorubicin in Estrogen Receptor-Positive Mammary Carcinoma
Source: Cancers (Basel). 2019 Oct 10;11(10):1528. doi: 10.3390/cancers11101528 (PMC6826976; doi:10.3390/cancers11101528)
Supplement: Supplementary file 1 [file cancers-11-01528-s001.pdf]

cancers

Supplementary Materials:

Inhibition of TFF3 Enhances Sensitivity- and Overcomes Acquired Resistance- to Doxorubicin in Estrogen Receptor-positive Mammary Carcinoma

Han Ming Poh, Yi Shiou Chiou, Qing Yun Chong, Ru-Mei Chen, Kanchugarakoppal S. Rangappa, Lan Ma, Tao Zhu, Alan Prem Kumar, Vijay Pandey, Basappa, Soo-Chin Lee and Peter E. Lobie

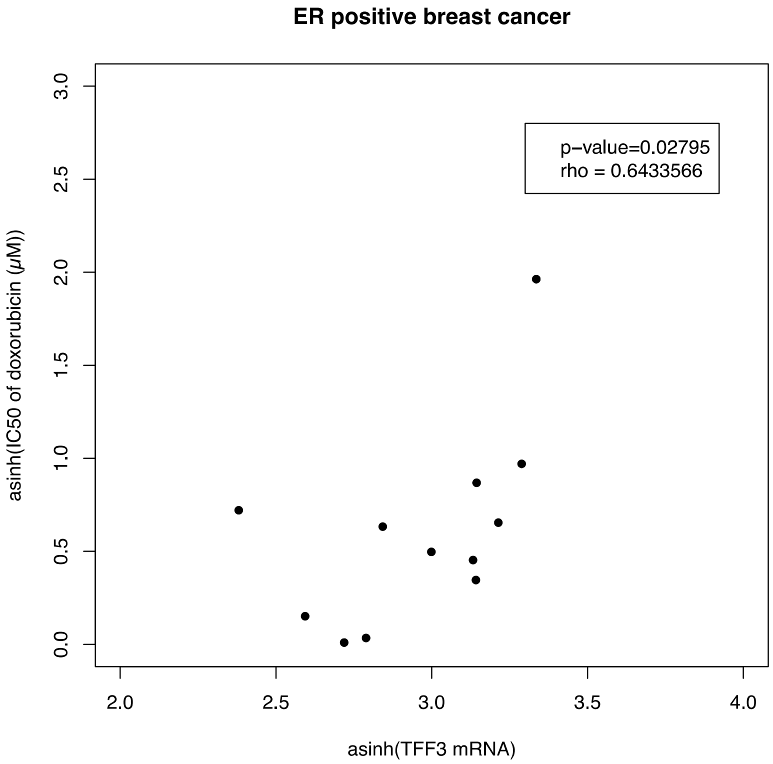

Figure S1. Spearman’s rank correlation between TFF3 mRNA expression and doxorubicin sensitivity in a panel of 12 ER+ MC cell lines.

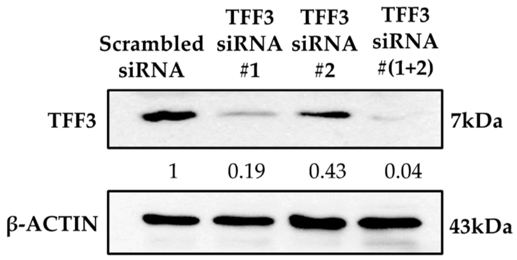

Cell-lines		Doxorubicin (nM)	
		IC_{50}	+SD
MCF-7	Scrambled siRNA	126.6	1.6
	TFF3 siRNA #1 (s277470)	89.6	5.2
	TFF3 siRNA #2 (s14041)	98.8	2.5
	TFF3 siRNA (Combined)	76.5	8.6

Figure S2. Combined use of two independent TFF3 siRNA exhibits greater effectiveness in enhancing cellular sensitivity towards doxorubicin. MCF-7 cells pre-incubated with 20nM of two independent TFF3 siRNA for 12 hours were treated with increasing doses of doxorubicin for 72 hours in monolayer culture. Cell viability was quantified using the AlamarBlue cell viability assay where the 50% inhibitory concentration (IC_{50}) values for doxorubicin were determined using GraphPad Prism 5. Western blot analysis for the protein expression of TFF3. β-ACTIN was used as input control. Band intensities were quantified by ImageJ and normalized to input control, where intensity ratio of scrambled siRNA treatment was set to 1.

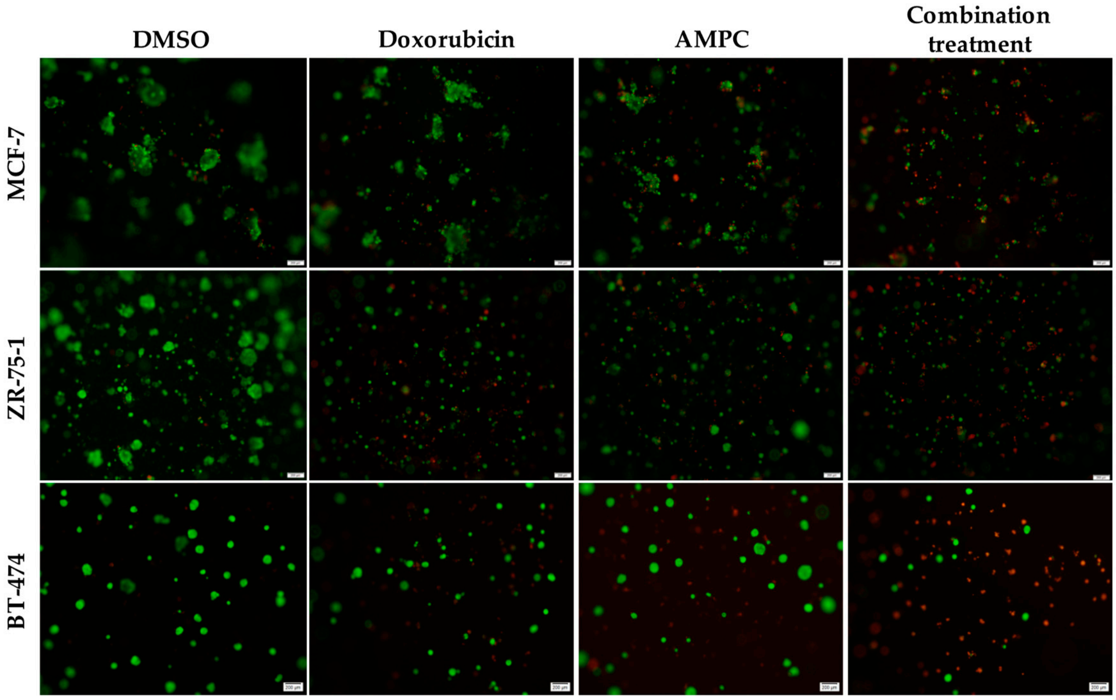

Figure S3. AMPC-mediated inhibition of TFF3 enhances doxorubicin-induced cell death. MCF-7, ZR-75-1 and BT-474 cells were pre-cultured in medium containing 5% FBS and 4% Matrigel for 5 days, followed by treatment with doxorubicin with or without AMPC for 9 days (MCF-7: 50nM dox, 2μM AMPC; ZR-75-1: 200nM dox, 5μM AMPC; BT-474: 50nM dox, 5μM AMPC). Cells were stained with LIVE/DEAD cell viability assay and visualized using florescence microscopy. The scale bar represents 200μm.

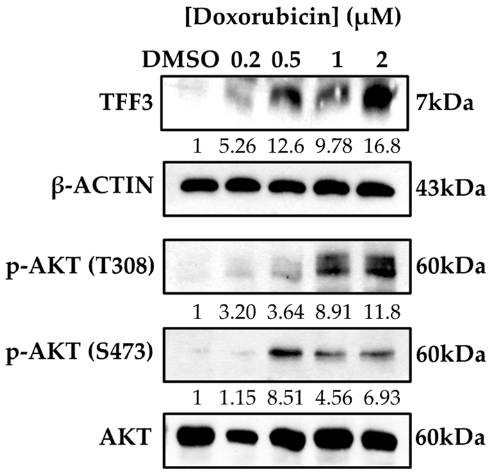

Figure S4. Doxorubicin induces TFF3 expression and AKT activation. MCF-7 cells in monolayer culture were treated with indicated doses of doxorubicin for 24 hours. Western blot analysis showed the protein expressions of TFF3 and activated AKT and the level of phosphorylated AKT. β-ACTIN was used as input control. Band intensities were quantified by ImageJ and normalized to input control/total proteins for phosphorylated proteins, where intensity ratio of vehicle DMSO treatment was set to 1.

(a) **(b)**

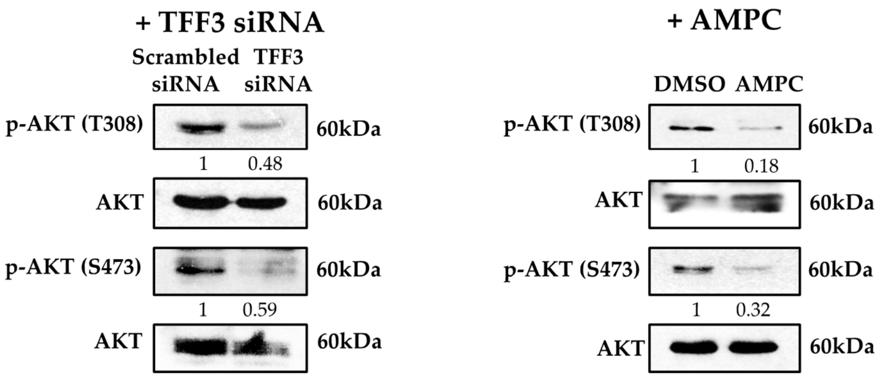

Figure S5. Depletion/inhibition of TFF3 decreases the activation of AKT. MCF-7 cells in monolayer culture were treated with **(a)** 20nM of two TFF3 siRNA combined overnight or **(b)**10μM AMPC for 24 hours. Western blot analysis showed the protein expressions of TFF3. β-ACTIN was used as input control. Band intensities were quantified by ImageJ and normalized to input control/total proteins for phosphorylated proteins, where intensity ratio of scrambled siRNA/vehicle DMSO treatment was set to 1.

(a) **(b)**

Cell Type	Doxorubicin (μM)	
	IC_{50}	±SD
Dox Control	137.45	37.12
Dox Resistant	561.0	52.47

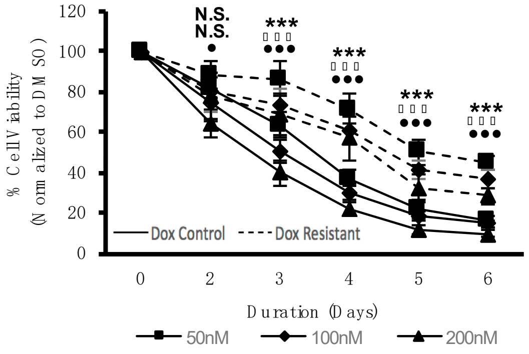

Figure S6. *Cont.*

(c)

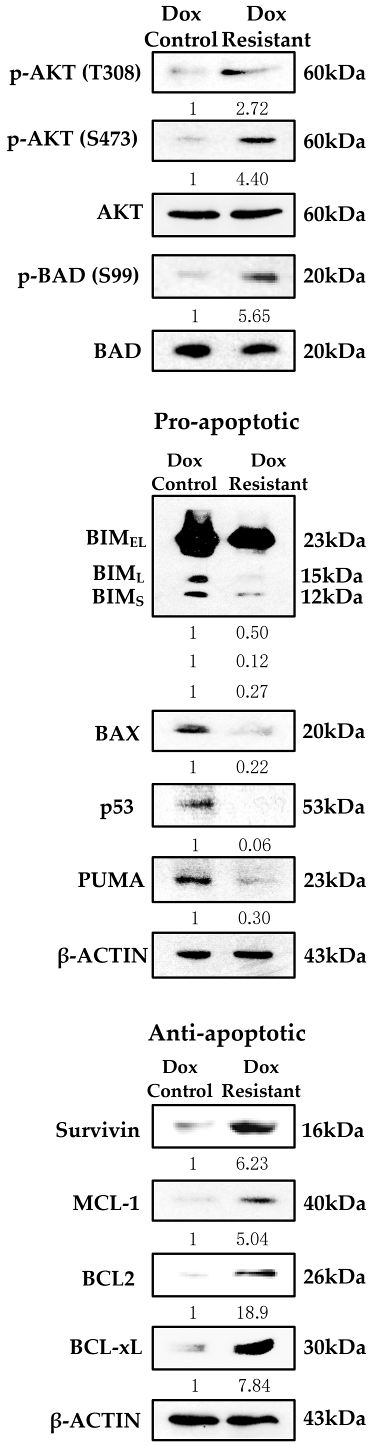

(d)

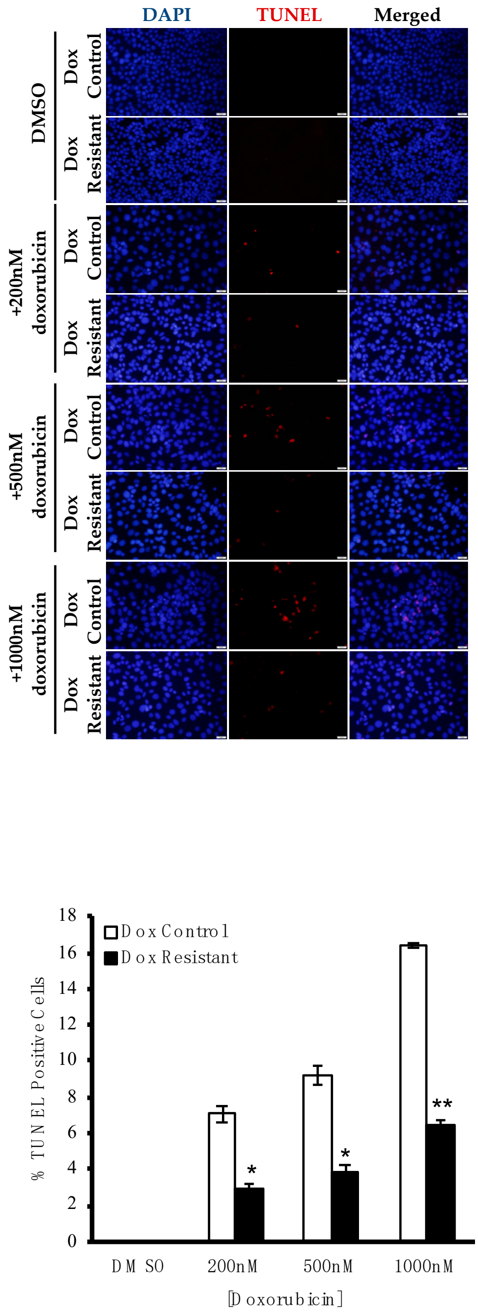

(e)

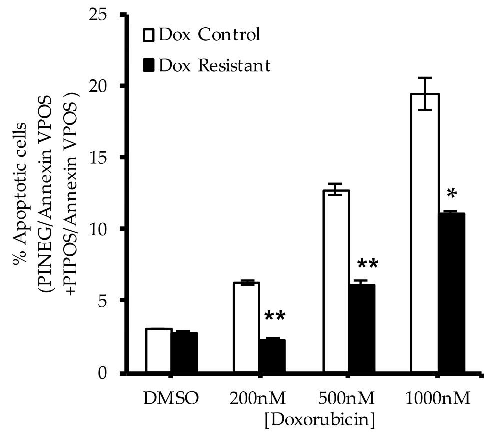

Figure S6. Characterization of acquired doxorubicin resistant MCF-7 cells. Control and doxorubicin resistant MCF-7 were treated with **(a)** increasing doses of doxorubicin for 72 hours; **(b)** indicated doses of doxorubicin over a period of 6 days in monolayer culture. **(c)** Western blot analysis determined the basal levels of AKT and BAD phosphorylation and expression of apoptotic-related proteins in control and doxorubicin resistant MCF-7 cells. β-ACTIN was used as input control. Band intensities were quantified by ImageJ and normalized to input control/total proteins for phosphorylated proteins, where intensity ratio of doxorubicin control cells was set to 1. **d-e.** Total apoptosis was analyzed in the control and doxorubicin resistant MCF-7 cells treated with indicated concentrations of doxorubicin for **(d)** 24 hours, followed by TUNEL staining and visualization by fluorescent microscopy; **(e)** 48 hours, followed by Annexin V-PI staining and quantification by flow cytometry. Cell viability was quantified using the AlamarBlue cell viability assay where the 50% inhibitory concentration (IC_{50}) values for doxorubicin were determined using GraphPad Prism 5. % of TUNEL positive cells was quantified by ImageJ. The scale bar represents 50μm. Bar charts show means ± standard deviations. *,◆ and ● denotes control VS doxorubicin resistant cells upon 50nM, 100nM and 200nM doxorubicin treatment respectively where * $P < 0.05$, ** $P < 0.01$ and *** $P < 0.001$ (Student's T Test).

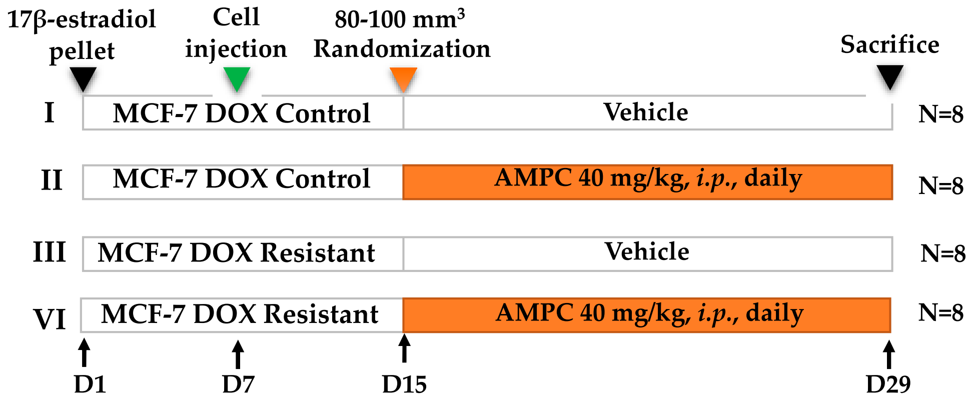

Figure S7. Study design for orthotopic mouse model. Eight days after implantation (with tumour size approximately 80-100 mm^3), the mice were randomized into 4 groups (n = 8) and injected intraperitoneally with vehicle or AMPC at 40 mg/kg body weight daily for 2 weeks.

(a)

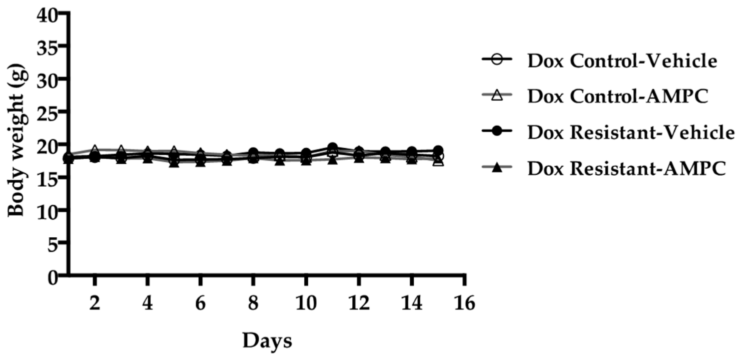

(b)

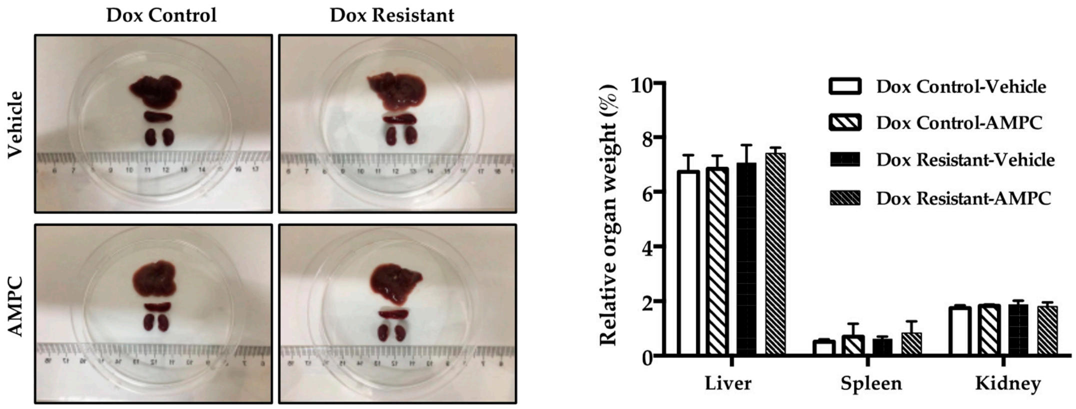

Figure S8. Changes in the body weights and appearances of vital organs of mice treated with AMPC. **(a)** Mice body weights were recorded daily after AMPC treatment. After sacrifice, **(b)** representative photographs of vital organs (liver, spleen and kidney) and relative organ weights (%) were measured. Relative organ weights (%)/organ body index = (organ weight × 100)/ body weight; AMPC was administered to mice at doses of 40 mg/kg; values are the mean ± SD (n=8).

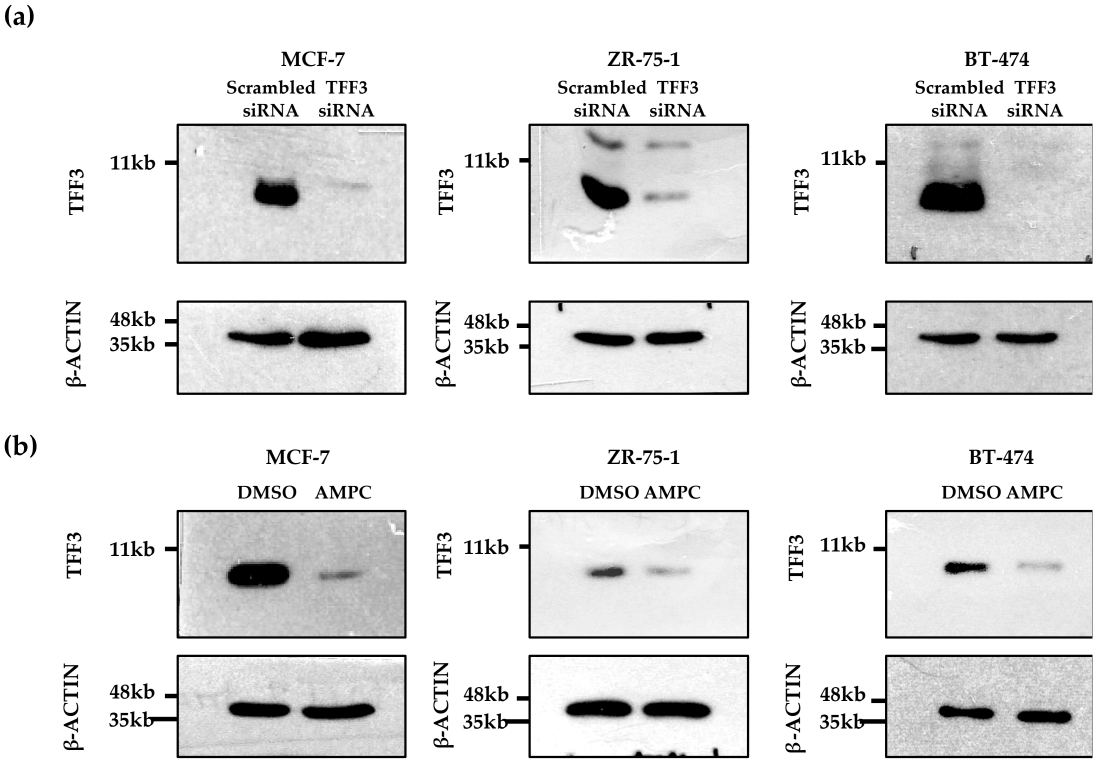

Figure S9. Blots from Figure 1a and b.

(a)

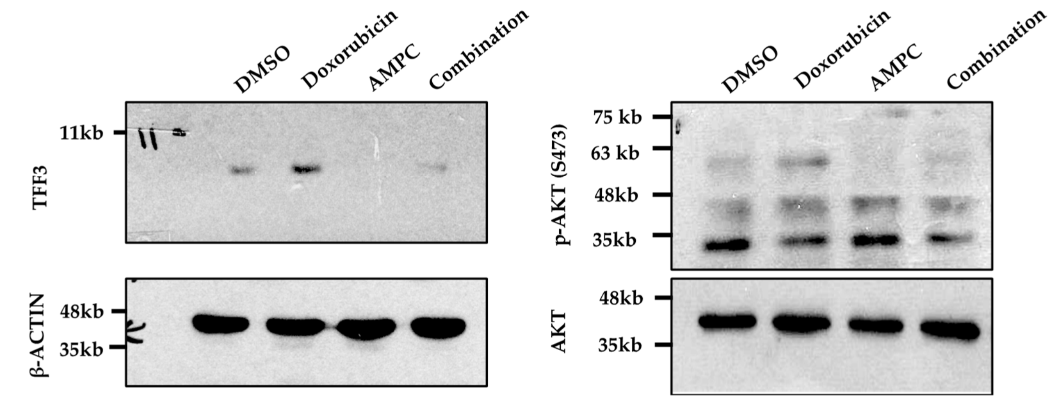

(b)

Pro-apoptotic

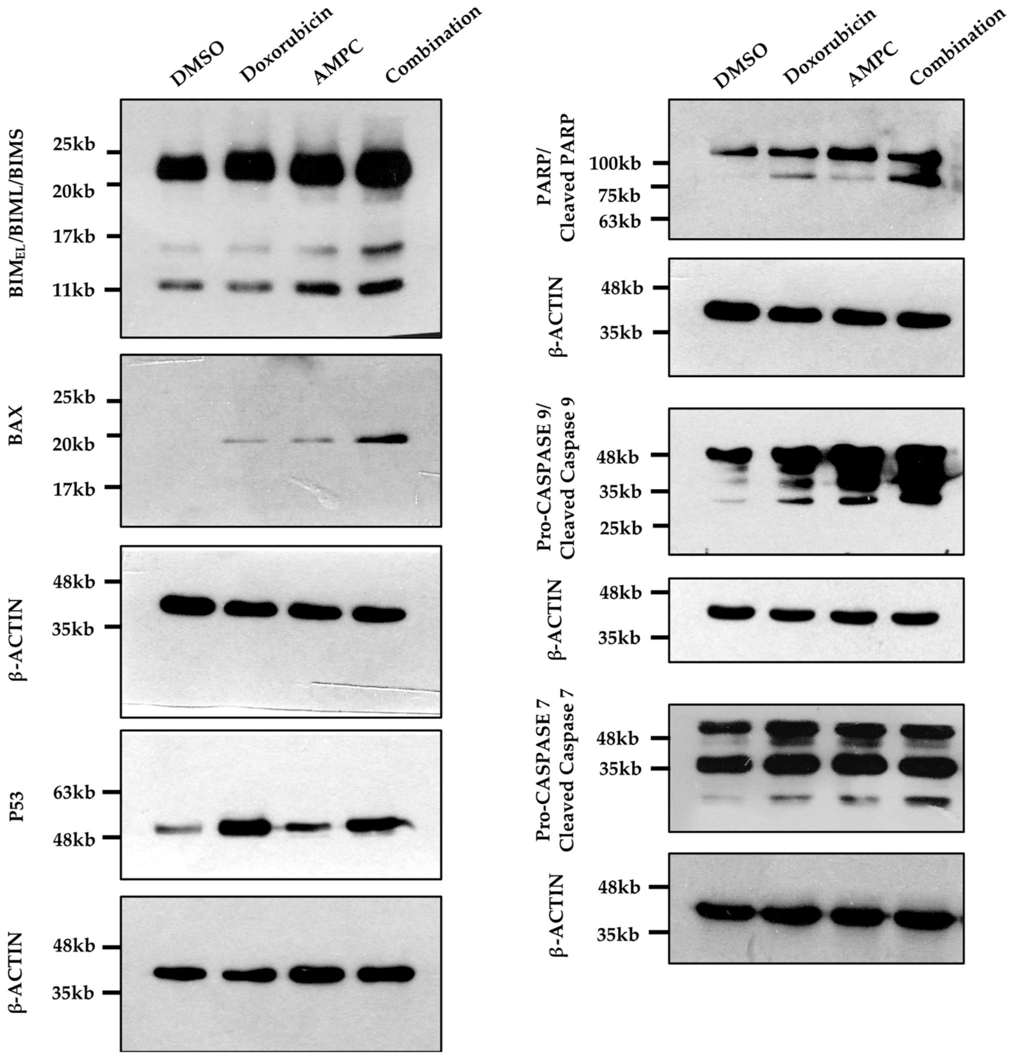

Figure S10. *Cont.*

Anti-apoptotic

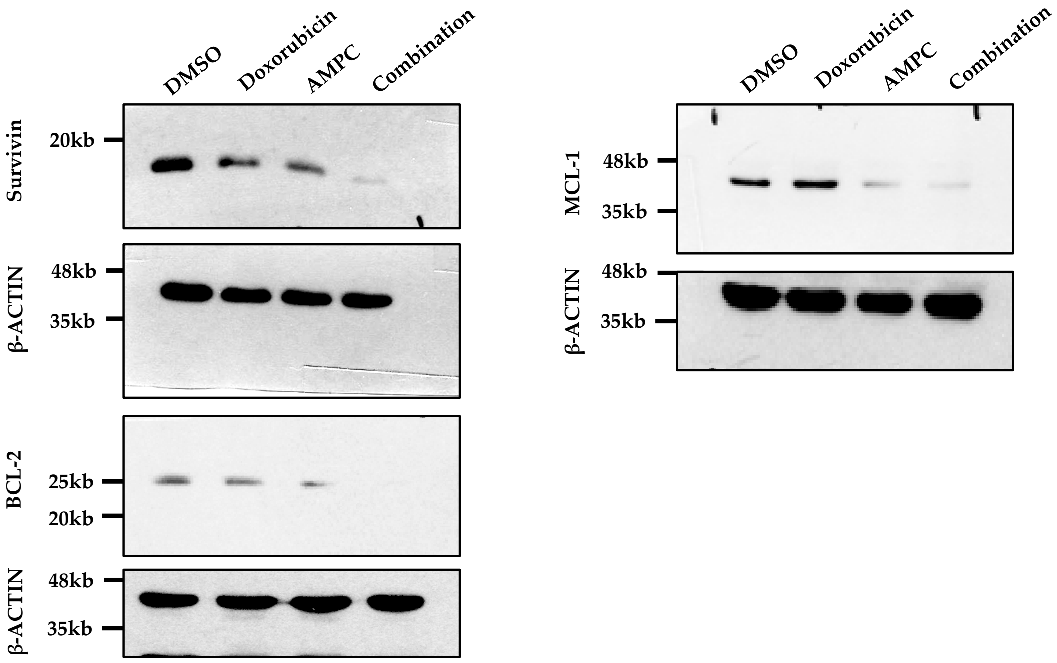

Figure S10. Blots from Figure 3a and b.

(b)

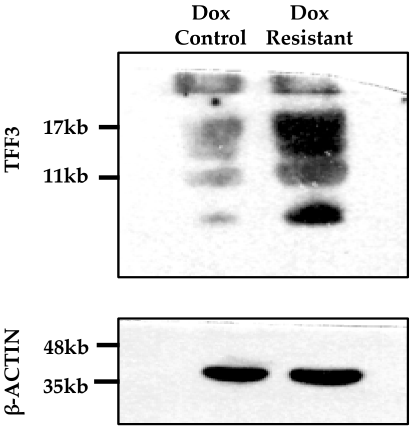

Figure S11. Blots from Figure 4b.

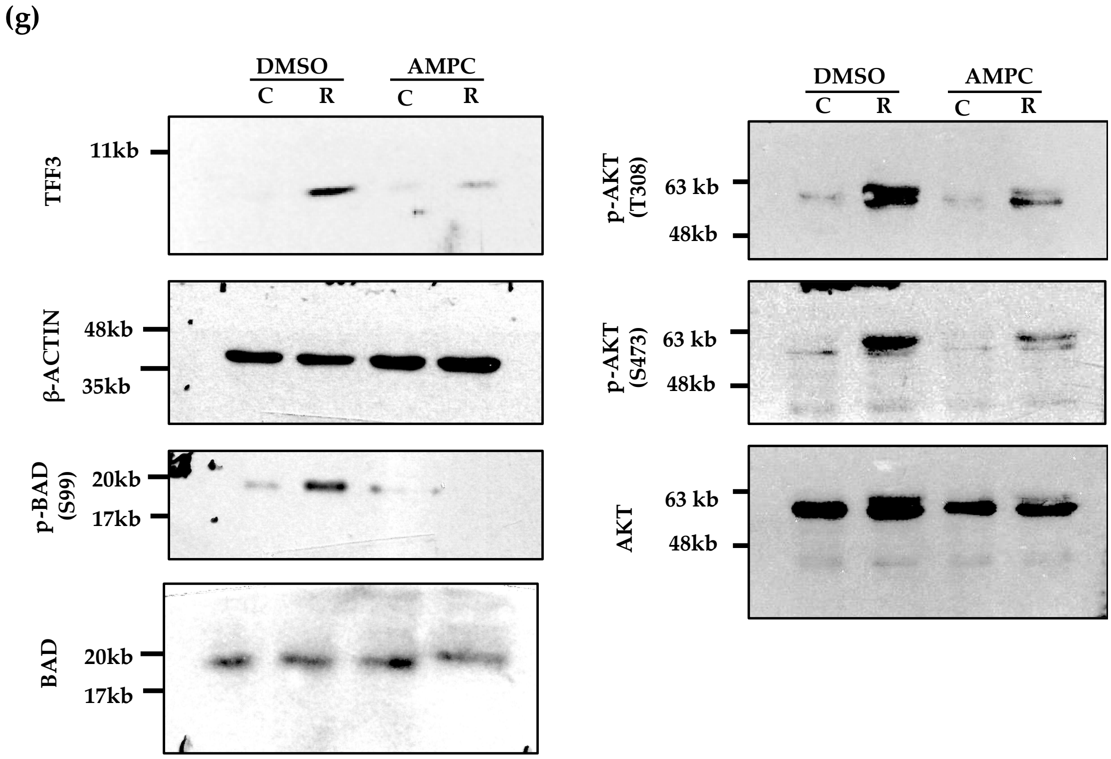

Figure S12. Blots from Figure 5g.

© 2019 by the authors. Submitted for possible open access publication under the terms and conditions of the Creative Commons Attribution (CC BY) license (http://creativecommons.org/licenses/by/4.0/).
